# Seaweed Growth Monitoring with a Low-Cost Vision-Based System

**DOI:** 10.3390/s23229197

**Published:** 2023-11-15

**Authors:** Jeroen Gerlo, Dennis G. Kooijman, Ivo W. Wieling, Ritchie Heirmans, Steve Vanlanduit

**Affiliations:** 1 InViLab Research Group, University of Antwerp, Groenenborgerlaan 171, 2020 Antwerp, Belgium; jeroen.gerlo@uantwerpen.be (J.G.); ritchie.heirmans@uantwerpen.be (R.H.); 2 Intelligent Autonomous Mobility Center, 5612 DX Eindhoven, The Netherlands; dennis@i-am.center; 3 Aqitec, 3311 RM Dordrecht, The Netherlands; ivo.wieling@aqitec.com

**Keywords:** aquaculture, seaweed monitoring, underwater stereo imaging, image segmentation

## Abstract

In this paper, we introduce a method for automated seaweed growth monitoring by combining a low-cost RGB and stereo vision camera. While current vision-based seaweed growth monitoring techniques focus on laboratory measurements or above-ground seaweed, we investigate the feasibility of the underwater imaging of a vertical seaweed farm. We use deep learning-based image segmentation (DeeplabV3+) to determine the size of the seaweed in pixels from recorded RGB images. We convert this pixel size to meters squared by using the distance information from the stereo camera. We demonstrate the performance of our monitoring system using measurements in a seaweed farm in the River Scheldt estuary (in The Netherlands). Notwithstanding the poor visibility of the seaweed in the images, we are able to segment the seaweed with an intersection of the union (IoU) of 0.9, and we reach a repeatability of 6% and a precision of the seaweed size of 18%.

## 1. Introduction

Cultivated seaweed, also referred to as macroalgae, may become an important source of alternative protein for sustainable food systems and global food security. Other benefits include the capture of carbon, absorption of excess nutrients, and restoration of ecosystems and biodiversity. Seaweed farming and harvesting are still very small-scale in Europe [1,2]. While the cultivation of seaweed in Asia has been practiced for decades, transferring this success to a European environment will require the development of dedicated automation [3,4]. Macroalgae production is almost entirely a maritime activity, and needs innovative equipment to increase productivity and quality. The inspection and monitoring of crops and infrastructure at sea are dangerous and expensive tasks, requiring boats and divers when performed manually. Remote and automated monitoring will significantly reduce labour costs and reduce the carbon emissions of vessels [5].

Remote satellite-based or airborne sensors can provide valuable data on plant growth [6,7,8,9,10]. The paper by Rowan et al. [6] gives an overview of different technologies for monitoring submerged aquatic vegetation (SAV). The authors conclude that remote sensing can be used to achieve vegetation classification accuracies of up to 99%. At the same time, they admit that a more granular level monitoring at the canopy level (as is needed for vertical seaweed farming) has been less explored. The literature on seaweed monitoring using remote sensing techniques is extensive, but most papers describe techniques that are not usable for seaweed that is growing vertically underwater. In the case of shallow water, [11] demonstrate that a multispectral camera installed on a drone can be used to determine the so-called normalized difference vegetation index (NDVI) of submerged seaweed in a lagoon. While they show that the NDVI can be used to monitor the seaweed growth over time in the lagoon, their method is limited to shallow water and hence it cannot be used for vertical seaweed farms (which are more common in Europe). Chen et al. [12] propose a seaweed monitoring technique based on RGB imaging. They show that classification accuracies between 70% and 85% can be obtained for different types of seaweed. Even though their results are valuable for seaweed farms in Asia, their method focuses on the determination of the so-called Above Ground Biomass (AGB), and hence is not usable for farms in Europe which are usually vertical farms. Other research on the monitoring of seaweed that is visible from a above the water show that high accuracies can be obtained. Diruit et al. [13] were able to classify seaweed on a rocky shore using a drone and a hyperspectral camera with an accuracy of 95%, while Chen et al. [14] successfully applies multispectral imaging and machine learning to detect seaweed in an intertidal zone.

While most papers in the literature focus on the inspection of seaweed from an aerial vehicle (a drone) we will focus in this paper on the monitoring from an underwater vehicle. Peres et al. [15] developed an underwater sensor and data acquisition system that is able to monitor light intensity, temperature, and plant motion. Their goal is to fix multiple instances of the sensor system to the seaweed lines. While this monitoring system enables farmers to make informed decisions, the sensor system cannot directly be used to estimate the physical size of the seaweed leaves. Other researchers use sonar sensors for seaweed monitoring. In [16], the authors report the design of a prototype sideband sonar sensor system that is able to follow the vertical lines in a seaweed farm. They have tested the system in a realistic simulation environment and on an underwater robot in a seaweed farm. The validation experiment of their system for plant growth monitoring will be performed in the future. Hamana et al. [17] demonstrated that a multibeam echosounder can be used to map the 3D structure of a seaweed forest (both canopy height and inter-individual distances of the plants were measured).

In this paper, we propose a vision-based sensor system for growth monitoring of a vertical seaweed farm. The use of cameras in an underwater environment is a challenging task, and development of these systems is either quite costly [18,19] or requires very dedicated assemblies [20]. We will demonstrate that low-cost consumer grade cameras (both RGB and stereo cameras) can be used to determine the size of the seaweed. While growth monitoring using underwater cameras has been successfully conducted on fish and mammals [21,22,23], to our knowledge, there are no studies in the literature on vision-based underwater seaweed growth monitoring.

Results in the laboratory [24] indicate that segmented camera images can be used to reliably estimate the size of different types of seaweed (correlation coefficients higher than 85% were obtained). In this paper, we will investigate if image-based growth monitoring can also be applied in a more realistic environment (in situ). We report the results of an experimental *Saccharina* plantation at the mouth of the River Scheldt estuary in The Netherlands. Saccharina latissima is a type of seaweed that is valued for human consumption. It can grow naturally along the coast of the north Atlantic Ocean, and is being cultivated in a test phase in several locations in the Netherlands. The cultivation methods used there are similar to those for other valued species of seaweed [25], making it a useful use case. Supported by machine learning, the collected images are sufficient to provide valuable information to the farmers. The combination of seaweed segmentation via neural networks and scale information via the stereo depth data can provide seaweed length estimates and a qualitative comparison between seaweed growth stages. This in turn can potentially eliminate the need for labor-intensive manual inspection.

The measurements and research described in this paper are conducted as part of the ICT-Agri-Food aUtomaTed Open PrecIsion fArming Platform (UTOPIA) project (https://utopia-project.eu/, (accessed on 15 May 2023)). Its goal is to enable farmers to implement precision farming with an affordable time and cost investment by providing them with an open data platform that interfaces with low-level mapping, planning and measurement technologies.

## 2. Materials and Methods

### 2.1. In Situ Setup

Our measurements were conducted on a crop of Saccharina latissima, commonly known as sugar kelp, cultivated in an artificial bay of the island *Neeltje Jans*. This is an island on the border of the North Sea and the Oosterschelde (a former estuary of the River Scheldt). The water is saline and affected by ebb and flow currents, but otherwise devoid of any sea or river currents. We have selected this type of seaweed because it is of high economic importance in Europe. It is harvested for various purposes, including food, feed stock and industrial applications. Sugar kelp can also be used for the production of biofuels, fertilizers, pharmaceuticals and cosmetics. Saccharina latissma plants consists of elongated ribbon-like brown-colored blades that can reach a length of several meters.

**Figure 1:**
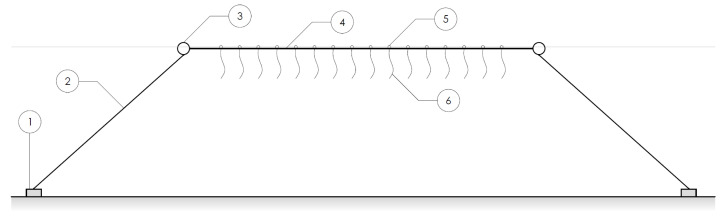
Common seaweed farm typology: Floaters or buoys (3) are held in position on the water surface using anchors (1) and tethers (2). A long rope line (4) is suspended between the fixed buoys. The seaweed (6) is seeded on this suspended line, growing downwards and flowing freely in the water current. Attached buoys and/or floaters (3, 5) keep the seaweed in the upper water column in order to catch sunlight.

Sprouts of seaweed are hung on rope lines between buoys, the distance between the buoys fixed at approximately 10 m with tethers. Under the weight of the plants, the rope lines curve underwater to a depth of one to two meters. Figure 1 shows a layout of the test setup in the early growth stage.

**Figure 2:**
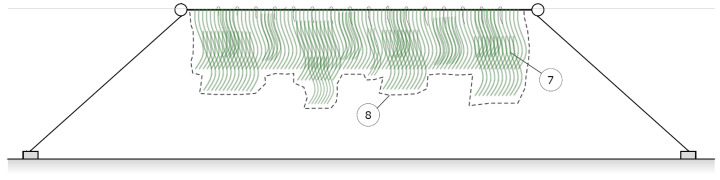
Seaweed farm schematic during growth. (7) Dense seaweed bundles; (8) delineated surface area of the seaweed, approximating it as a continuous 2D sheet.

The seaweed is left to grow between January and May, growing vertically downwards from just a few centimeters to up to 4 m. As the seaweed grows, it becomes densely packed, forming an almost continuous bundle down and along the seeded line (see Figure 2). Due to the geometry of the farm setup, most of the seaweed growth will occur within a 2D plane formed by the suspended rope line and a combination of gravity and the water currents. For our monitoring efforts, we therefore choose to approximate the seaweed as flat sheets hanging along the underwater lines. By measuring the size of these sheets, an estimate of farm yield can be acquired.

While this design is developed to consist of hundreds of these suspended lines within one farm, the experimental setup at Neeltje Jans consisted of two lines placed in parallel, with a total line length of approximately 20 m.

### 2.2. Methodology

The goal of our method is to track the potential yield of the seaweed plants using only visual data. With only visuals, it is not possible to measure the seaweed mass directly. Using the sheet approximation as described above, we aim to provide an estimate of the seaweed plant size as it grows underwater.

**Figure 3:**
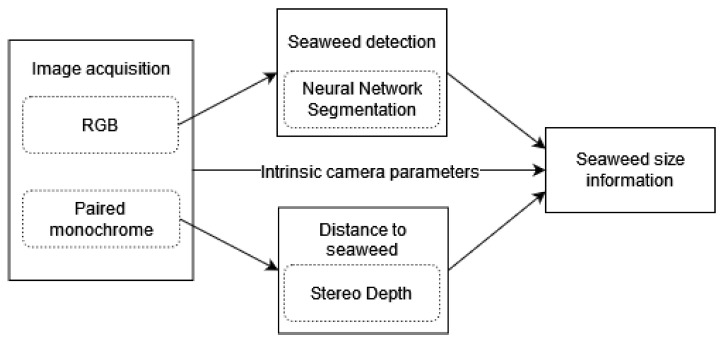
Sequence of the automated monitoring process. The separate components are designed to be integrated in the UTOPIA framework.

A breakdown of the process is shown in Figure 3. First, we acquire underwater images, described in Section 2.3. Section 2.4 describes the detection of seaweed using RGB images. Section 2.5 explains the processing of the monochrome image pairs to a distance estimate of the seaweed sheets, providing crucial scale information. Combining the results from both components allows the calculation of a seaweed yield estimate.

### 2.3. Image Acquisition

**Figure 4:**
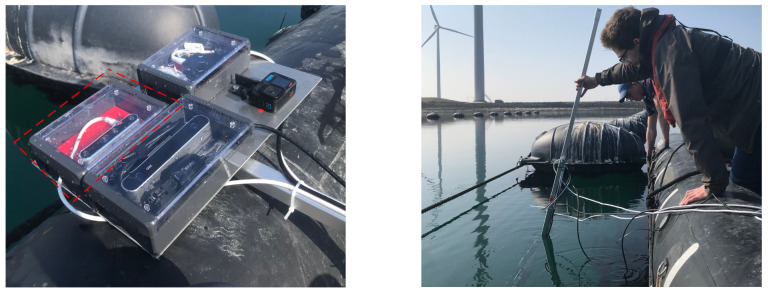
(**Left**) Area within the red dashed line shows the waterproof housing and mounting of the Realsense camera. (**Right**) The camera setup submerged and recording during one of the measurement campaigns at Neeltje Jans.

Since the seaweed grows vertically downwards (in wide sheets), the optimal angle to monitor them is from the side, perpendicular to their growth direction. This requires the imaging equipment to be placed underwater. Collecting quantitative scale measurements is also crucial to conduct growth monitoring; for this, we chose to use a stereo range camera. The Intel Realsense D455 is a compact consumer stereo camera, with an RGB camera included within its design. The D455’s stereo baseline of 95mm gives it an advantage on triangulating distances beyond 3 m accurately, compared to other compact stereo cameras [26,27]. These factors allow for a relatively easy and low-cost assembly of a waterproof housing to collect stereo and RGB images underwater. Figure 4 shows the setup in the field. In addition to the Realsense stereo camera, we also used GoPro 5 camera to obtain high-quality RGB recordings of the environment. We noticed that the image quality (in terms of contrast) of the GoPro camera was substantially better than the RGB images of the Realsense. However, in the Realsense camera, the coordinate system of the RGB camera is geometrically calibrated with the monochrome cameras that are used to determine the distance to the seaweed. Therefore, we decided to use the Realsense RGB camera for image segmentation instead of the GoPro. Our setup also included a ZED 1.0 stereo camera (with a larger baseline of 110 mm compared to the Realsense stereo camera). Because the sensitivity of ZED camera images was too low in the underwater environment, we decided not to use the ZED camera in this paper.

To keep our method available to use with other camera setups, we chose not to use the processed depth data provided by the onboard Realsense processor. The monochrome stereo image pair is instead captured and stored, then processed independently with an open-source algorithm (see Section 2.5). We use the calibration parameters that are given by the camera manufacturer [28]. In general, an adapted calibration procedure should be used for 3D underwater imaging [29]. We circumvent the need for this relatively complex calibration by placing the cameras in the housing such that they are orthogonal to the housing and water interfaces, thereby undergoing no refraction.

**Figure 5:**
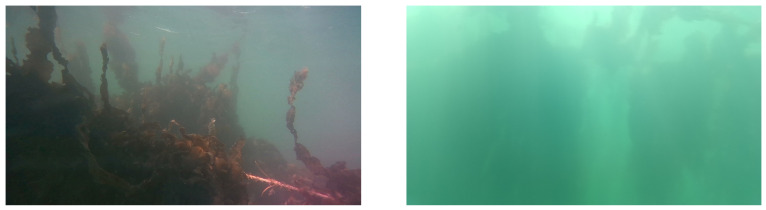
(**Left**) Sharp images of seaweed in the foreground, but too close to determine plant size. (**Right**) The seaweed plant is in frame, but is too far away to be sufficiently sharp due to the haze created by turbidity and lighting conditions at the moment of measurement.

The images were recorded with a frame rate of one set of images (RGB image and grayscale image pair) per second. In total, 800 image sets were recorded while the boat continuously sailed parallel to one of the lines at a distance of about 2 m from the lines. The sample frequency (of one set per second) was chosen to obtain an overlap of at least 20% between the recorded image sets. Furthermore, we also performed a measurement of 100 image sets while the boat was in a stationary position. This was done to evaluate the repeatability of our procedure. With the camera setup correctly aimed and secure underwater, there are still other issues that stand in the way of collecting viable images of the growing seaweed. Because the seaweed grows in shallow open water, the turbidity can be quite high. This means images become hazy when the distance between the camera and the target increases, as light scatters off of floating particles in the water column. A shorter distance between the camera and the plants will mean a higher-quality image. However, taking close-up images of the seaweed does not provide usable data for our monitoring plan: we need the plant to be fully within frame to be able to determine its size. In practice, this means that the top and bottom of the plants needs to be in frame. This will allow the camera to limit itself to only horizontal movement when collecting images for a complete seaweed line. The Realsense D455 has a vertical viewing angle of 65${}^{\circ }$ with its RGB camera, and is therefore able to obtain a 4 m long vertically growing seaweed plant in frame at a minimum distance of 2.6 m. Figure 5 shows an example of images at badly selected distances, being either too close to provide useful information, or too far to distinguish the seaweed through the haze.

**Figure 6:**
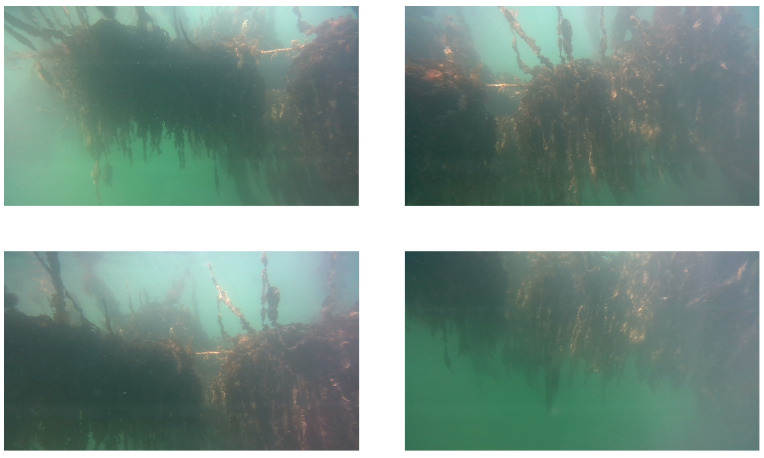
Example of underwater images taken with the Realsense RGB camera. Seaweed plants can take on different colors and shapes depending on current lighting conditions, depth and position within the seaweed bundles.

Over the course of two measurement days, 800 images were selected that had the seaweed in frame and were sufficiently sharp. Since the seaweed cultivation happens close to the surface, the currents and turbidity of the water can be heavily affected by weather conditions. Measurement campaigns were carried out during clear weather and calm seas, increasing visibility. Due to surface waves and the manual handling of the camera equipment, the image sequence was not taken from a steady camera angle. Figure 6 shows examples of underwater images taken by the RGB camera.

### 2.4. Seaweed Detection—Neural Network

#### 2.4.1. Challenges

With our underwater camera correctly aimed and recording, the next step was to distinguish and delineate the seaweed plants within our image. To achieve this, we relied on machine vision, more specifically on CNN segmentation algorithms. As already visible in Figure 6, segmenting the seaweed within these images poses significant challenges:

- Over a short sequence of images, and also within a single image itself, lighting conditions can vary wildly. This will make recognition based on color or brightness difficult, if not impossible. Strands of seaweed that grow deep underwater or densely together become shapeless and dark in the images.
- Support cables and other types of rigging can get covered in algae and other unwanted plant matter, which can closely resemble the actual seaweed crops. Ideally, these elements will be ignored by the segmentation algorithm.
- Due to the larger layout of the seaweed farm, a second line of seaweed will sometimes be visible behind the plants in the foreground of the image. Because of the difference in distance, seaweed on this second line has a discrete reduction in sharpness compared to the foreground plants.

#### 2.4.2. Annotation

**Figure 7:**
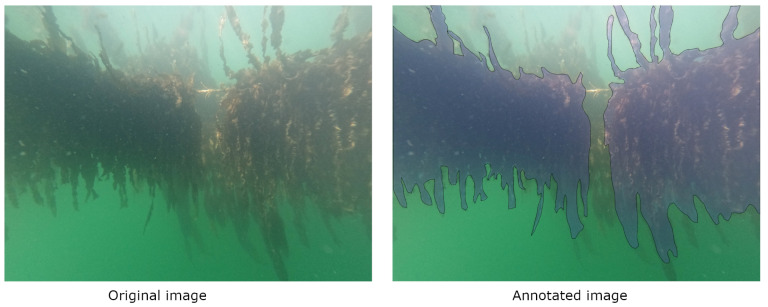
Example of manual annotation on a seaweed RGB image. (**Left**): original image. (**Right**): original image with the annotation superimposed in blue with a black border. Two bundles of seaweed are visible in the foreground, both growing on the closest rope line. Another bundle of seaweed is visible in the background, much more obscured by the water haze.

Automated detection requires a manual basis. To that end, 35 RGB images were annotated in detail using CVAT’s (Computer Vision Annotation Tool) polygon selection. These 35 images were chosen for maximum variety in orientation, lighting, sharpness and visibility out of the 800-image data set. During annotation, the choice whether to include blurry, dark or hazy sections of the image was often difficult. We chose to always select the seaweed that was clearly part of the closest line of seaweed, while leaving seaweed on any lines further back out of the annotation (see Figure 7). In this way, the calculation of a yield estimate for the image only includes the seaweed line that the camera is aimed at at that time.

#### 2.4.3. Segmentation Algorithm and Hyperparameters

The DeeplabV3+ segmentation architecture is an improved variant of the DeepLab semantic segmentation algorithm [30]. The Deeplab algorithm uses spatial pyramid pooling in its interconnected layer, which makes it successful at multi-scale feature detection [31]. This is useful in finding sections of seaweed in images, independently of its stage of development. The modifications made to DeeplabV3 enable the use of encoders and decoders, which give sharper object boundaries and clearer classification, and allow us to use pre-trained models. DeepLabV3+ is known for its accuracy in segmenting objects in images. It employs atrous convolution (also known as dilated convolution) and a deep neural network to capture fine details and make accurate pixel-wise predictions, which is crucial in underwater image segmentation where objects may be partially obscured or have intricate shapes and textures. In contrast to other deep learning-based image segmentation methods, DeepLabV3+ can produce cleaner and more well-defined object boundaries, which is particularly important in underwater imagery, where the distinction between objects and their surroundings can sometimes be challenging due to water turbidity and lighting conditions.

**Figure 8:**
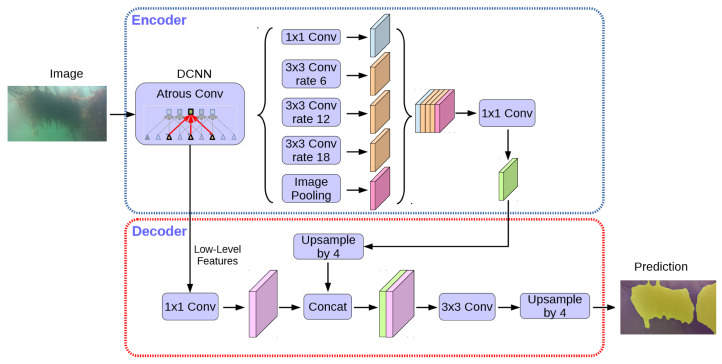
DeeplabV3+ architecture. The structure of the Atruous Spatial Pyramid Pooling (ASSP) decoder module is shown, followed by a simple decoder to acquire image prediction. Adapted from [31].

Figure 8 illustrates an overview of the network architecture, and we refer to the source texts for a more in-depth discussion.

The training of the model took place in a Python environment, supported by the open-source Pytorch library. As part of the the training, images are augmented [32] to introduce additional variety and effectively creating a larger dataset for training. Augmentations included resizing, mirroring, scaling and cropping, as well as adding blur, Gaussian noise and sharpening, plus changes to brightness, contrast and hue saturation.

To tune the computational performance and the quality of the segmentation, we varied the key hyperparameters for this model:

- *Image resolution*: Input resolutions of 256 × 256, 320 × 320 and 576 × 576 pixels were tested. A higher resolution during training will always improve the model quality, but is computationally costly.
- *Number of epochs*: This parameter denotes the number of complete passes through the training dataset. Using a higher epoch count consequently increases training time. The training was run at a standard setting of 100 epochs. Increasing the number of epochs to 200 showed no change to the model on any resolution setting.
- *Batch size*: The batch size determines how many image samples are processed before the model is updated. The batch size is recommended to be a power of 2, matching the hardware of the processing unit. We tested batch sizes of 4 and 16 at 256 × 256 and 320 × 320 pixels. The 576 × 576 pixel resolution could not be trained with a batch size of 16 due to hardware limitations.

#### 2.4.4. Training Results

**Figure 9:**
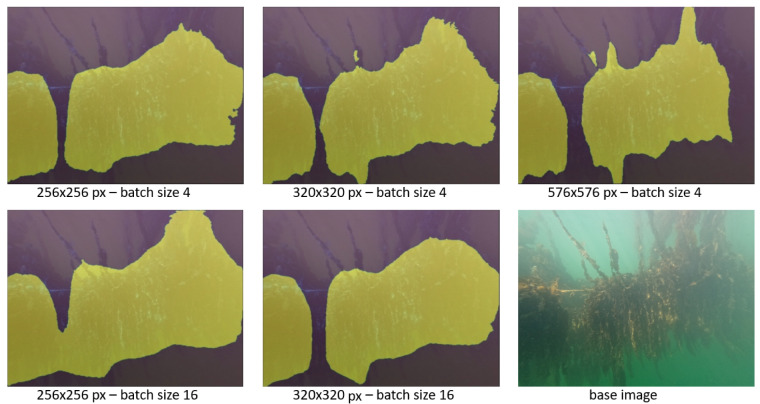
Segmentation results of the DeeplabV3+ model for different hyperparameters. Segmentation masks are shown as a yellow overlay, with the base image in the bottom right.

**Figure 10:**
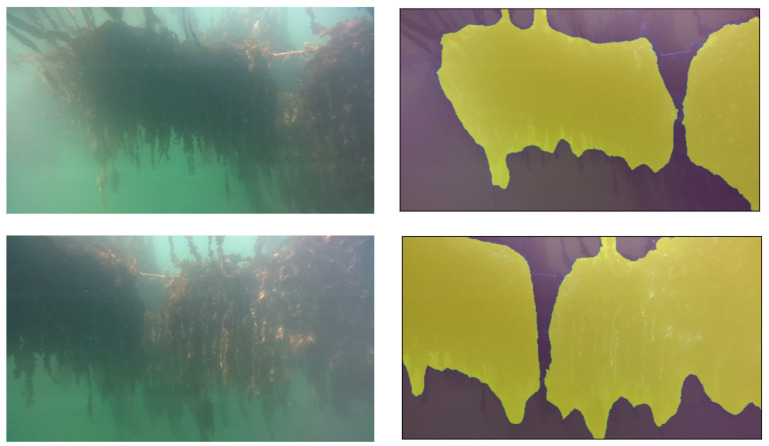
More examples of seaweed segmentation with the DeeplabV3+ model using our optimal hyperparameters. The model correctly distinguishes a gap between the seaweed plants in the foreground, and ignores the seaweed on a second line in the background. Finer details of trailing seaweed at the bottom of the plants is not detected due to limitations on pixel resolution for the segmentation.

Figure 9 shows the segmentation results for the different hyperparameter settings of the model. Training at the highest resolution allows the segmentation mask to be more detailed, while the difference caused by changing batch size does not offer a clear advantage. More examples of segmentation with the “576 px—batch size 4” model are shown in Figure 10. Aside from varying the hyperparameters, the training data was passed to the network with different assignments to the training or validation set. Each time, 28 images were used as training data, with 7 annotated images remaining to validate the performance of the model in each training step. Depending on this split, images in the validation set contain seaweed sections that are generally more hazy or more sharp. This results in the final model favoring to either detect or ignore hazy areas of seaweed.

**Figure 11:**
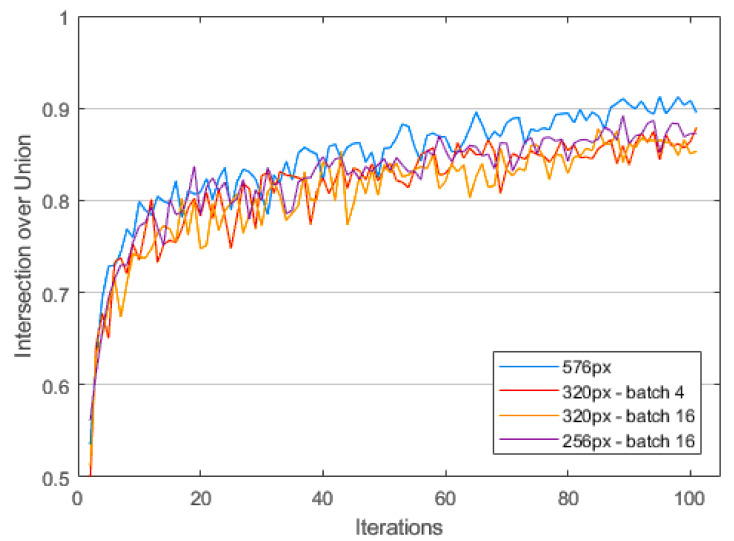
Evolution of the intersection over union (IoU) metric of the training images using different hyperparameters. A perfect model would reach an IoU of 1. The 576 px model converges towards an IoU of 0.9 after 100 iterations (epochs).

**Figure 12:**
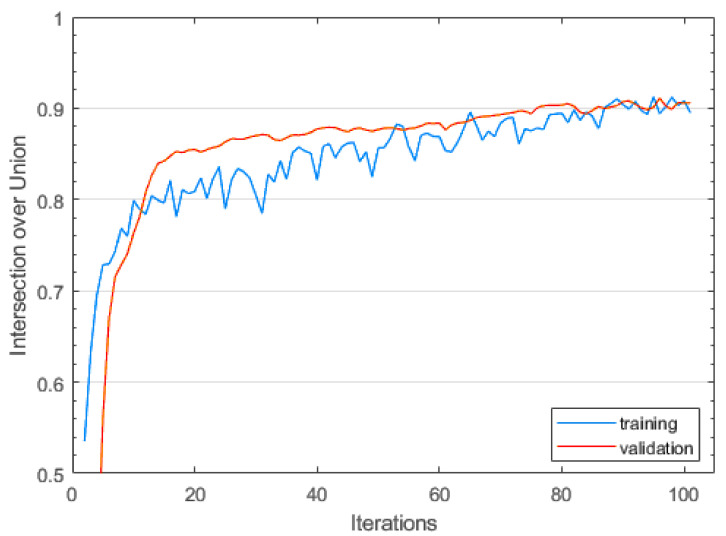
Intersection over union metric for the DeeplabV3+ model for seaweed segmentation. Training and validation IoU are approximately identical after 100 epochs.

The intersection over union (IoU) of training and validation images is a common metric to assess model quality in a quantifiable way. Figure 11 shows the evolution of the training IoU for the different hyperparameters, with the 576 px resolution model consistently giving the best result. Out of 10 training runs, we selected the optimal result. With an IoU of 0.9 and training and validation approximately equal, we can assume to be safe from overfitting (see Figure 12).

#### 2.4.5. Robustness Test

Due to the nature of the in situ setup, we do not have the ability to measure a ground truth for the seaweed surface area. Since this precludes the validation of the detected values of seaweed surface area via a direct comparison, we need to rely on statistics-based validation. Therefore, we employ the following test.

In theory, a perfect segmentation model would give identical results over a series of images where the camera and seaweed is perfectly still. As part of the in situ measurements, the imaging equipment was kept at a stationary position as much as possible for 1 min, taking 100 RGB images of the same seaweed plant section. By studying the variation of the segmented surface area within this image series, we obtain an estimate of the in situ precision of the model performance.

**Figure 13:**
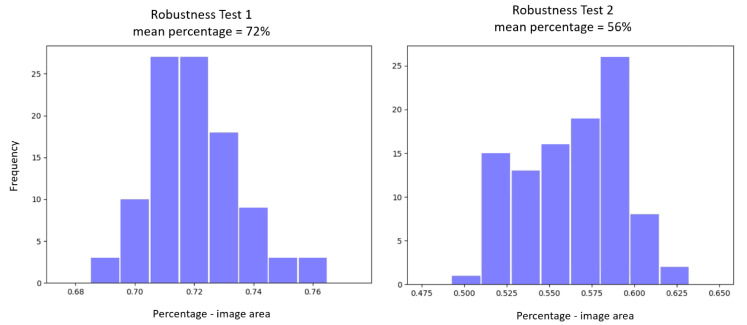
Spread of the segmented surface area as a percentile of the total image, for two one-minute steady camera positions. There are no large outliers in either test.

We conducted this test two times: on the first test, on average 72% of the image was detected as seaweed, with a variation of ±4%. On the second test, this was 56%, with a variation of ±6%. During each one-minute test, neither the camera nor the seaweed were perfectly still. Surface waves influence the position and angle of the camera, while water currents cause slight changes in occlusion and clumping of the seaweed strands. The variation in the test results is therefore a combination of actual changes to the surface area during the time of the test, combined with perturbations caused by the precision of the detection. Figure 13 shows the spread of the detections for both tests in a histogram. Both series of images are evenly spread around the mean, and they have similar deviation. Moving forward with our method, we will consider the error on the seaweed segmentation for a single image to be 6% or less.

### 2.5. Distance Estimation

**Figure 14:**
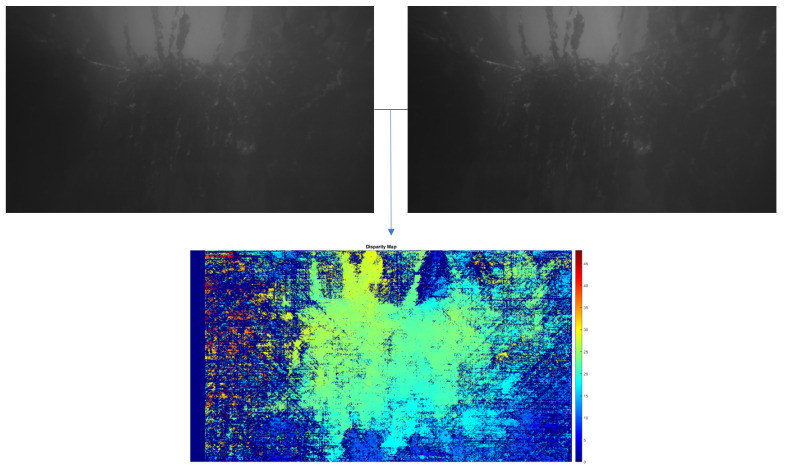
Construction of a disparity map. (**top**) Original left and right stereo images. (**bottom**) Normalized disparity map, calculated via the Semi-Global Block Matching algorithm.

By determining the range of the camera to the seaweed plant, we can calculate the actual scale of pixel distances and areas in our images. This range can be estimated via triangulation with a stereo camera. Using the disparity between a pair of stereo images to determine range is a well-studied problem, we refer to the literature [33,34,35,36] for a more in-depth description. Figure 14 shows an example of a pair of stereo images and its disparity map.

We use the Semi-Global Matching algorithm described by Hirschmuller et al. [37] to calculate a disparity map between two stereo images. The algorithm works by computing an aggregated cost by summing the costs to reach pixel with disparity from each direction. The Python OpenCV library provides a modified version of the algorithm, allowing the use of pixel-block matching instead of single-pixel matching. This speeds up the stereo matching significantly, depending on the chosen block size (we selected a block size of 16).

The resulting disparity image is then converted into real-world range information via triangulation. The calibration parameters of the stereo camera setup provide the necessary information to calculate the triangulation step:

(1) Z=b*f/d,

with depth *Z*, stereo baseline *b*, camera focal point *f* and disparity *d* [38]. Both the camera focal point and the baseline were obtained from the camera datasheet. With the range to objects in our images known, the actual size of an object can be approximated. However, the disparity map does not return an errorless result. For our underwater seaweed images, accurately matching points in a pair is hindered by several factors:

- Lack of distinctive features in the image pair;
- Noise caused by floating particles in the water column;
- General image blur caused by water diffraction;
- Flashes of light and darkness caused by surface waves;
- Low lighting.

This combination of noise, blur and the varying lighting of features in the seaweed bundles can cause significant variability in the calculated range for single image points. At the same time, having the range for each single pixel in an image is not useful information. That is because in our setup, we approximate the seaweed plants as large, flat sheets, thereby already ignoring the variation in thickness of the bundles of seaweed.

We choose to average out the smaller range fluctuations by taking a 2D median over the central area of our image (600 × 400 px). The disparity map is most accurate in the center of the image, and avoids effects from lens distortion. By using this median, sections of the stereo image that show open water or that are occluded by floating particles will be mostly ignored. The resulting value will give a good estimate for the range to the segmented seaweed surface area for that image.

#### Range Measurement Precision

Out of water, distance measurements with the Realsense D445 are shown to have good precision and accuracy [26]. With standard settings, the Realsense camera projects a structured infrared dot pattern to assist with stereo matching of image pairs. For the underwater measurements, this IR projection was disabled. Tests of the camera without this projection showed no noticeable change in accuracy or precision at distances beyond 2 m.

**Figure 15:**
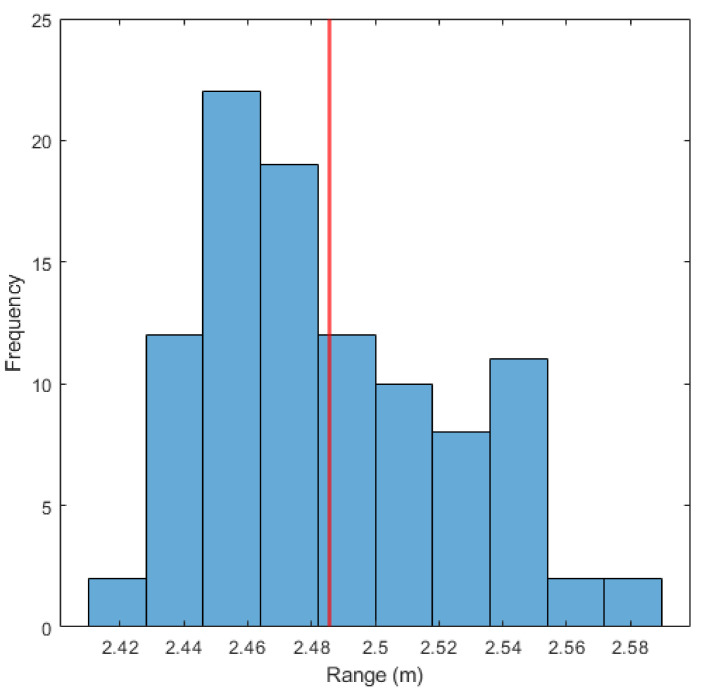
Spread of the distance measurement 2D median with a steady plant target, calculated for 100 images. The red line denotes the mean.

Due to the nature of the in situ setup, conducting alternative reference measurements of the distance between our camera and the seaweed was not possible without employing divers or more specialized equipment. To verify the robustness of our distance estimation, we conducted a similar test as for the seaweed segmentation. The imaging equipment was steadied as much as possible for 1 min, taking 100 stereo images of the same seaweed plant section. The distance to the plant was then calculated for each image, using the 2D median over the image as described above. Figure 15 shows a histogram of the resulting ranges. The results are evenly distributed, with a variation of ±0.11 m over the 100 images. (see Figure 15).

## 3. Results

**Figure 16:**
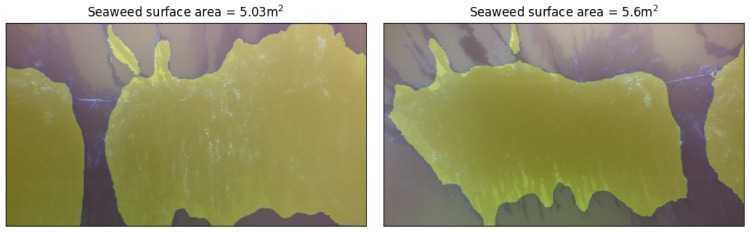
Segmented images, with seaweed crop yield in square meters as calculated by our algorithm. The pixel area of seaweed on the left image is larger than the right, but the left image was captured at a shorter distance from the seaweed (1.99 m vs. 2.97 m). Our method returns a smaller surface area in m${}^{2}$ for the left image.

**Figure 17:**
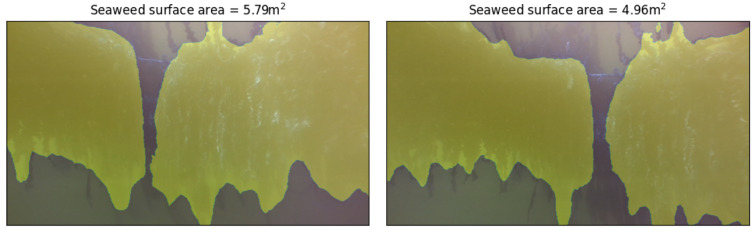
Additional examples of segmented images, with seaweed crop yield in square meters as calculated by our algorithm.

For every image set (RBG image and grayscale stereo pair) out of the 800 recorded samples, we have used the DeeplabV3 model to perform the image segmentation of the RBG images (as described in Section 2.4). From the segmented images we can calculate the size of the seaweed in pixels squared. At the same time, we use for every image set the grayscale stereo pair to evaluate the (average) distance between the camera and the seaweed plant (see Section 2.5). Using this distance information we can convert the seaweed size from pixels squared to meters squared. This provides a functional yield in m${}^{2}$ seaweed for every image. In Figure 16, we illustrate that the seaweed size increases as we move further away from the lines. This is obvious because a larger field of view is covered in that case. In order to estimate the yield of the complete seaweed lines, we would need to combine the yield for every separate image set (and correct for the overlap in the different images which is visible in the two segmented images shown in Figure 17). This process was not part of the scope of the current paper. Based on the average segmentation accuracy of MoU=0.9 (see Section 2.4.4) and the estimated precision on the distance of ±0.11 m (or 4.4% relative precision), we can calculate that the error on the yield size is about 0.9 m${}^{2}$ (or about 18% relative error). In our calculations, we assumed that the seaweed can be be approximated as a flat sheet (which is a fairly good assumption for sugar kelp, but which might not hold for other types of seaweed).

## 4. Discussion

The D455 Realsense stereo camera was able to acquire clear and usable images to monitor seaweed growth. Haze, blur and refractions due to the underwater conditions significantly worsen the quality of stereo matching, while the slow movement of seaweed provides the opportunity to collect multiple images for fast averaging. This makes the technique still effective at providing valuable data to farmers. The collected images were also clear enough to train a segmentation algorithm that is able to recognize seaweed. It is able to distinguish the cultivated *Saccharina* from fouling and support lines. However, these images were taken during nearly perfect weather conditions with a high underwater visibility. For monitoring in less ideal conditions, it will be important to balance out underwater haze, and the range to the seaweed to keep it in view. Developing de-hazing techniques will play a vital role to keep the visual monitoring of seaweed viable in more turbid water. Linking haziness during the segmentation with depth measurement will also improve the viability of underwater stereo monitoring.

Because our method approximates the seaweed plants as flat sheets, the resulting calculation of seaweed yield is presented as a surface area in m${}^{2}$. While this is a strong simplification, it helps keep our method robust with respect to the challenging underwater conditions. The value m${}^{2}$ is not directly usable as a measure of crop yield, but it can be used as a quantitative comparison between sections of the seaweed farm. If combined with the data of seaweed mass after a harvest, it can provide a predictive estimate of crop yield in kilograms for future cultivation cycles.

While the images collected for this research still required manual handling of the camera setup, the design principle assumes it to be attached to a remote surface vessel in future measurement campaigns. Compared to a completely submerged vehicle, a remote surface vessel has significant advantages with respect to ease of operation, communication and cost.

Our method is based on a deep learning network to segment underwater images of brown kelp alone (hence, it is biased toward this seaweed type). In the case our approach would be used for other seaweed types, it should be re-trained using relevant images (a transfer learning approach can be used in that case).

Our image-based seaweed yield estimation method can easily be integrated on a low-cost underwater vessel. As such, regular yield measurements can be performed at an affordable cost, which can provide farmers with detailed information about the growth rate their crops, allowing them to make informed decisions about their harvests. Accurate yield estimates can also assist seaweed companies to expand and diversify their products. For example, the production of biofuels from seaweed requires large quantities of biomass, which can be obtained through accurate yield estimates.

In conclusion, yield estimates can revolutionize the seaweed industry by providing farmers with accurate and real-time information about their crops, reducing waste, and enabling the production of new and innovative products.

## Data Availability

Data are contained within the article.

## References

[1] Addamo A., Calvo Santos A., Guillén J., European Commission, Directorate General for Maritime Affairs and Fisheries, Joint Research Centre (2022). The EU Blue Economy Report 2022.

[2] FOA (2022). Brief to The State of World Fisheries and Aquaculture 2022.

[3] Ahmed Z.U., Hasan O., Rahman M.M., Akter M., Rahman M.S., Sarker S. (2022). Seaweeds for the sustainable blue economy development: A study from the south east coast of Bangladesh. Heliyon.

[4] Campbell I., Macleod A., Sahlmann C., Neves L., Funderud J., Øverland M., Hughes A.D., Stanley M. (2019). The Environmental Risks Associated With the Development of Seaweed Farming in Europe—Prioritizing Key Knowledge Gaps. Front. Mar. Sci..

[5] Bostock J., Lane A., Hough C., Yamamoto K. (2016). An assessment of the economic contribution of EU aquaculture production and the influence of policies for its sustainable development. Aquac. Int..

[6] Rowan G.S.L., Kalacska M. (2021). A Review of Remote Sensing of Submerged Aquatic Vegetation for Non-Specialists. Remote Sens..

[7] Sagawa T., Mikami A., Aoki M.N., Komatsu T., Frouin R., Ebuchi N., Pan D., Saino T. (2012). Mapping Seaweed Forests with IKONOS Image Based on Bottom Surface Reflectance. Proceedings of the Remote Sensing of the Marine Environment II.

[8] Malthus T.J., Mishra D.R., Ogashawara I., Gitelson A.A. (2017). Bio-optical Modeling and Remote Sensing of Aquatic Macrophytes. Bio-optical Modeling and Remote Sensing of Inland Waters.

[9] Tonion F., Pirotti F. (2022). Seaweed Presence Detection Using Machine Learning And Remote Sensing. Int. Arch. Photogramm. Remote Sens. Spat. Inf. Sci..

[10] Uhl F., Bartsch I., Oppelt N. (2016). Submerged Kelp Detection with Hyperspectral Data. Remote Sens..

[11] Taddia Y., Russo P., Lovo S., Pellegrinelli A. (2020). Multispectral UAV monitoring of submerged seaweed in shallow water. Appl. Geomat..

[12] Chen J., Li X., Wang K., Zhang S., Li J., Sun M. (2022). Assessment of intertidal seaweed biomass based on RGB imagery. PLoS ONE.

[13] Diruit W., Le Bris A., Bajjouk T., Richier S., Helias M., Burel T., Lennon M., Guyot A., Ar Gall E. (2022). Seaweed Habitats on the Shore: Characterization through Hyperspectral UAV Imagery and Field Sampling. Remote Sens..

[14] Chen J., Li X., Wang K., Zhang S., Li J. (2022). Estimation of Seaweed Biomass Based on Multispectral UAV in the Intertidal Zone of Gouqi Island. Remote Sens..

[15] Peres C., Emam M., Jafarzadeh H., Belcastro M., O’Flynn B. (2021). Development of a Low-Power Underwater NFC-Enabled Sensor Device for Seaweed Monitoring. Sensors.

[16] Stenius I., Folkesson J., Bhat S., Sprague C.I., Ling L., Özer Ö., Bore N., Cong Z., Severholt J., Ljung C. (2022). A System for Autonomous Seaweed Farm Inspection with an Underwater Robot. Sensors.

[17] Hamana M., Komatsu T. (2021). Mapping 3D structure of a *Sargassum* forest with high-resolution sounding data obtained by multibeam echosounder. ICES J. Mar. Sci..

[18] Kunz C., Singh H. (2008). Hemispherical refraction and camera calibration in underwater vision. Proceedings of the OCEANS 2008.

[19] Sedlazeck A., Koch R. Calibration of Housing Parameters for Underwater Stereo-Camera Rigs. Proceedings of the British Machine Vision Conference 2011.

[20] Dadios E.P., Almero V.J., Concepcio R.S., Vicerra R.R.P., Bandala A.A., Sybingco E. (2022). Low-Cost Underwater Camera: Design and Development. J. Adv. Comp. Intell. Intell. Inf..

[21] Lai P.C., Lin H.Y., Lin J.Y., Hsu H.C., Chu Y.N., Liou C.H., Kuo Y.F. (2022). Automatic measuring shrimp body length using CNN and an underwater imaging system. Biosys. Eng..

[22] Yu J., Zhou Y., Guo Y., Li Z., Ren Y., Li L., Dong Y. (2022). Effects of air replenishers on the growth and body morphology of four fish species in an underwater aquaculture system. Aquaculture.

[23] Morisaka T., Sakai M., Hama H., Kogi K. (2022). Body length and growth pattern of free-ranging Indo-Pacific bottlenose dolphins off Mikura Island estimated using an underwater 3D camera. Mamm. Biology.

[24] Alves-Lima C., Azevedo Teixeira A.R., Hotta C.T., Colepicolo P. (2019). A cheap and sensitive method for imaging *Gracilaria* (Rhodophyta, Gracilariales) growth. J. Appl. Phycol..

[25] Lucas J.S., Southgate P.C. (2012). (Eds.) Aquaculture.

[26] Heinemann M., Herzfeld J., Sliwinski M., Hinckeldeyn J., Kreutzfeldt J. A metrological and application-related comparison of six consumer grade stereo depth cameras for the use in robotics. Proceedings of the 2022 IEEE International Symposium on Robotic and Sensors Environments (ROSE).

[27] Keselman L., Woodfill J.I., Grunnet-Jepsen A., Bhowmik A. Intel(R) RealSense(TM) Stereoscopic Depth Cameras. Proceedings of the 2017 IEEE Conference on Computer Vision and Pattern Recognition Workshops, CVPR Workshops.

[28] Carfagni M., Furferi R., Governi L., Santarelli C., Servi M., Uccheddu F., Volpe Y. (2019). Metrological and Critical Characterization of the Intel D415 Stereo Depth Camera. Sensors.

[29] Digumarti S.T., Taneja A., Thomas A., Chaurasia G., Siegwart R., Beardsley P. Underwater 3D Capture using a Low-Cost Commercial Depth Camera. Proceedings of the 2016 IEEE Winter Conference on Applications of Computer Vision (WACV 2016).

[30] Chen L.C., Zhu Y., Papandreou G., Schroff F., Adam H. Encoder-Decoder with Atrous Separable Convolution for Semantic Image Segmentation. Proceedings of the European Conference on Computer Vision (ECCV).

[31] Chen L.C., Papandreou G., Schroff F., Adam H. (2017). Rethinking Atrous Convolution for Semantic Image Segmentation. arXiv.

[32] Choi H., Park J., Yang Y.M. (2022). A Novel Quick-Response Eigenface Analysis Scheme for Brain–Computer Interfaces. Sensors.

[33] Scharstein D., Szeliski R. (2002). A taxonomy and evaluation of dense two-frame stereo correspondence algorithms. Int. J. Comp. Vis..

[34] Bleyer M., Gelautz M. (2005). A layered stereo matching algorithm using image segmentation and global visibility constraints. ISPRS J. Phot. Remote Sens..

[35] Seitz S., Curless B., Diebel J., Scharstein D., Szeliski R. A Comparison and Evaluation of Multi-View Stereo Reconstruction Algorithms. Proceedings of the 2006 IEEE Computer Society Conference on Computer Vision and Pattern Recognition (CVPR’06).

[36] Kamencay P., Breznan M., Jarina R., Lukac P., Radilova M. (2012). Improved Depth Map Estimation from Stereo Images Based on Hybrid Method. Radioengineering.

[37] Hirschmuller H. (2008). Stereo Processing by Semiglobal Matching and Mutual Information. IEEE Trans. Patt. Anal. Mach. Intell..

[38] Du Y.C., Muslikhin M., Hsieh T.H., Wang M.S. (2020). Stereo Vision-Based Object Recognition and Manipulation by Regions with Convolutional Neural Network. Electronics.

